# Normalisation to Blood Activity Is Required for the Accurate Quantification of Na/I Symporter Ectopic Expression by SPECT/CT in Individual Subjects

**DOI:** 10.1371/journal.pone.0034086

**Published:** 2012-03-21

**Authors:** Peggy Richard-Fiardo, Philippe R. Franken, Audrey Lamit, Robert Marsault, Julien Guglielmi, Béatrice Cambien, Fanny Graslin, Sabine Lindenthal, Jacques Darcourt, Thierry Pourcher, Georges Vassaux

**Affiliations:** 1 INSERM U948, Biothérapies Hépatiques, CHU Hôtel Dieu, Nantes, France; 2 CHU de Nantes, Institut des Maladies de l'Appareil Digestif, Nantes, France; 3 Centre Antoine Lacassagne, Nice, France; 4 Faculté de Médecine, Université de Nice Sophia-Antipolis, Nice, France; 5 Laboratoire TIRO, UMRE 4320, iBEB, DSV, Commissariat à l'Energie Atomique, Nice, France; University of Texas, M.D. Anderson Cancer Center, United States of America

## Abstract

The utilisation of the Na/I symporter (NIS) and associated radiotracers as a reporter system for imaging gene expression is now reaching the clinical setting in cancer gene therapy applications. However, a formal assessment of the methodology in terms of normalisation of the data still remains to be performed, particularly in the context of the assessment of activities in individual subjects in longitudinal studies. In this context, we administered to mice a recombinant, replication-incompetent adenovirus encoding rat NIS, or a human colorectal carcinoma cell line (HT29) encoding mouse NIS. We used ^99m^Tc pertechnetate as a radiotracer for SPECT/CT imaging to determine the pattern of ectopic NIS expression in longitudinal kinetic studies. Some animals of the cohort were culled and NIS expression was measured by quantitative RT-PCR and immunohistochemistry. The radioactive content of some liver biopsies was also measured *ex vivo*. Our results show that in longitudinal studies involving datasets taken from individual mice, the presentation of non-normalised data (activity expressed as %ID/g or %ID/cc) leads to ‘noisy’, and sometimes incoherent, results. This variability is due to the fact that the blood pertechnetate concentration can vary up to three-fold from day to day. Normalisation of these data with blood activities corrects for these inconsistencies. We advocate that, blood pertechnetate activity should be determined and used to normalise the activity measured in the organ/region of interest that expresses NIS ectopically. Considering that NIS imaging has already reached the clinical setting in the context of cancer gene therapy, this normalisation may be essential in order to obtain accurate and predictive information in future longitudinal clinical studies in biotherapy.

## Introduction

The sodium iodide symporter (NIS) is an integral membrane glycoprotein that mediates the uptake and concentration of iodide into cells. This protein is mainly expressed in the thyroid and stomach [1], although expression has been detected at other anatomical locations [2], [3]. In gene and cell therapies, ectopic NIS expression, associated with relevant radioisotopes such as ^123^I^−^, ^124^I^−^ or ^99m^TcO_4_^−^, has been used extensively in rodent models to monitor the efficacy of gene transfer using various imaging modalities (PET and SPECT) [1], [4]. With this technology, it is possible to evaluate the patterns of gene expression allowed by different gene delivery vectors [5], [6], [7], [8], [9], [10], [11], [12] or to assess the specificities of particular promoters [13], [14], [15], [16]. More recently, this methodology has been used to monitor the fates of cells with therapeutic potential which have been genetically-modified to express NIS [17], [18], [19], [20], [21]. In addition, it is possible to combine the imaging and therapeutic potentials of NIS to optimise radionuclide administration in cancer gene therapy [22], [23]. The status of NIS as a relevant reporter gene for SPECT imaging in cancer gene therapy has been validated recently in phase 1 clinical trials using conditionally replicating adenovirus [24], [25], although vector design and dose are critical to the successful visualisation of transgene expression [26].

The question of whether NIS imaging can provide quantitative information regarding transgene expression levels has been addressed in different experimental models: PET imaging of liver transduction using a ^124^I^−^ radiotracer [27], SPECT imaging of tumour transduction using ^123^I^−^ or ^99m^TcO_4_^−^ radiotracers [28], [29], [30] and SPECT imaging of cardiac gene therapy using a ^123^I^−^ radiotracer [21] were used to validate the methodology. Altogether, these studies demonstrated a good correlation between radiotracer accumulation and NIS gene and protein expression levels, as well as between radiotracer uptake measured by imaging and direct counting of the radioactivity associated with biopsies. There is, however, no consensus on how to present the data. Some authors have expressed their results as %ID/g (percentage of injected dose per gram of tissue) [21], [27], [28], while others have presented their data as a ratio of radiotracer accumulation in the transduced organ versus that in non-transduced tissues, taken as background [29]. The latter has the advantage of accounting for background values and is therefore less likely to be susceptible to individual experimental artefacts or variations. This point is particularly important as, unlike other reporter gene systems developed for PET or SPECT imaging, the amount of radiotracer localised in NIS-expressing tissues varies depending on its blood concentration. In addition, when ^131^I^−^ is used to treat differentiated thyroid carcinomas in humans, it is known that uptake to the target tissue, and thus treatment success, is more dependent on the blood concentration than on administered activity [31], [32]. There is, thus, a theoretical risk of artefactual variability. Considering that the monitoring of gene expression and transfer using NIS has been validated in patients [24], [25], and that the images obtained are used to evaluate the feasibility of ^131^I radioiodine therapy through dosimetric determinations [25], a full assessment of the methodology is required, particularly in the context of the assessment of activities in individual subjects in longitudinal studies, where data cannot be smoothed by a statistical analysis.

In the present work, we examined this issue using SPECT/CT and ^99m^TcO_4_^−^ as radiotracer in kinetics spanning more than sixty days after NIS gene transfer. More specifically, we determined whether quantitative data presented as %ID/g or %ID/cc provide a correct assessment of ectopic NIS transgene expression or whether blood-normalised-uptake (BNU) corrections are necessary to obtain a coherent dataset.

## Results

### SPECT/CT imaging and immunohistochemistry of NIS expression upon systemic injection of Ad-CMV-rNIS

**Figure 1:**
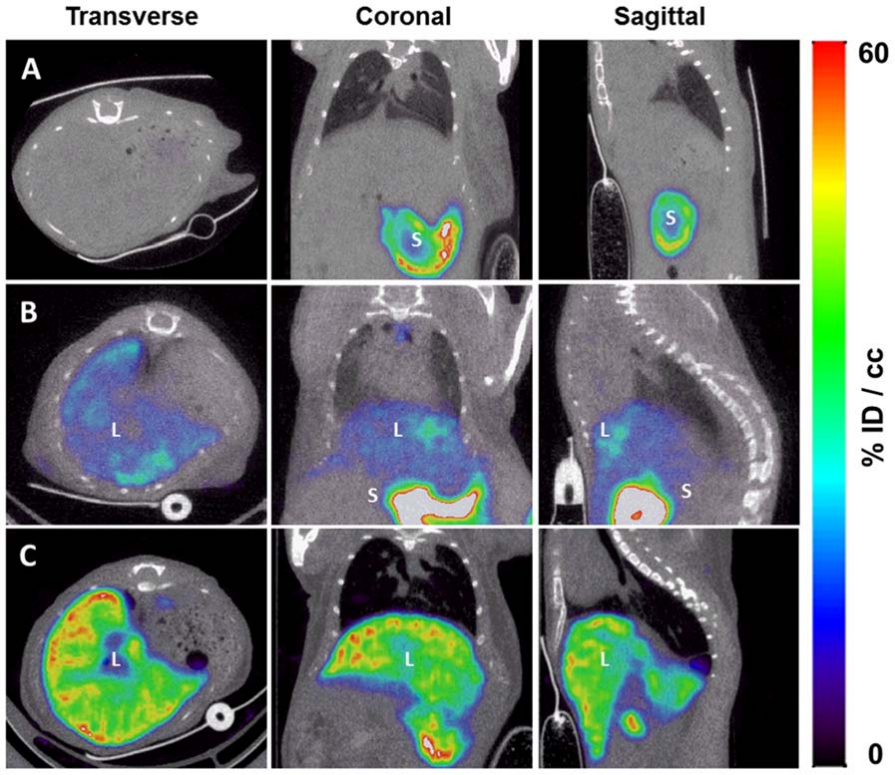
Imaging of NIS expression by microSPECT-CT. Transverse, coronal and sagittal slices from SPECT images of Balb/c mice intravenously injected with (A) saline buffer (n = 4), (B) 5×10^8^ PFU (n = 3) or (C) 1×10^9^ PFU Ad-CMV-rNIS (n = 3). Forty-eight hours later, mice received an intraperitoneal injection of 100 MBq ^99m^TcO_4_- and were imaged with a microSPECT-CT camera (eXplore speCZT, General Electric). (S) stomach and (L) liver.

**Figure 2:**
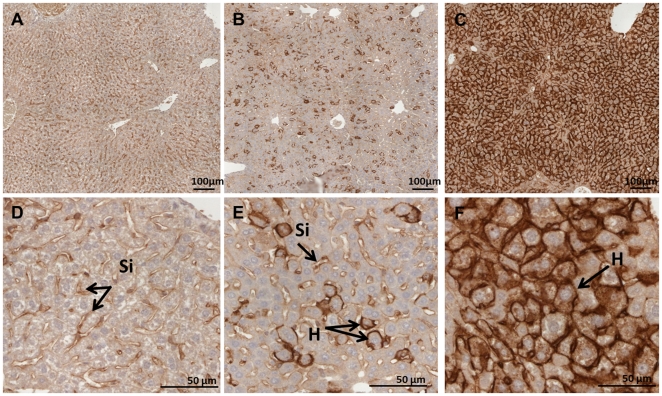
Immunohistochemical staining of NIS protein in adenovirus-transduced livers. Representative NIS immunostaining of liver sections from control (panels A and D) and Ad-CMV-rNIS-injected mice (panels B and E: 5×10^8^ PFU, panels C and F: 1×10^9^ PFU). Livers taken from one animal per condition were processed. Magnification: see bars on panels. Si: sinusoidal capillaries, H: hepatocytes.

To visualise gene transfer, Ad-CMV-rNIS doses varying from 5×10^8^ to 1×10^9^ PFU were injected systemically to Balb/c mice. Serial SPECT/CT scans were performed, upon injection of the ^99m^TcO_4_^−^ radiotracer up to 60 d after virus administration. Fig. 1 shows representative, selected coronal, transverse and sagittal views obtained 2 d after the administration of saline buffer (Fig. 1A) or 5×10^8^ (Fig. 1B) or 1×10^9^ PFU (Fig. 1C) Ad-CMV-rNIS. As expected from previous studies [33], [34], significant activity was noted in the liver when the animals were injected with 1×10^9^ PFU virus, as a result of ectopic NIS expression in this organ. Transgene expression was very significantly lower when 5×10^8^ PFU were administered (Figs. 1B versus 1C). In both cases, the activity was detectable from 5 h post virus administration (not shown). In addition, radiotracer accumulation was observed in the spleen 24 h after virus administration but this uptake was only transient and disappeared rapidly (not shown). Immunohistological staining of liver biopsies of these animals confirmed the dramatic difference observed by SPECT/CT in NIS expression (Fig. 2). In the animals administered with 1×10^9^ PFU Ad-CMV-rNIS, more than 90% of hepatocytes showed NIS-specific immunoreactivity (Figs. 2C and 2F).

### Measurement of blood and muscles activity

**Figure 3:**
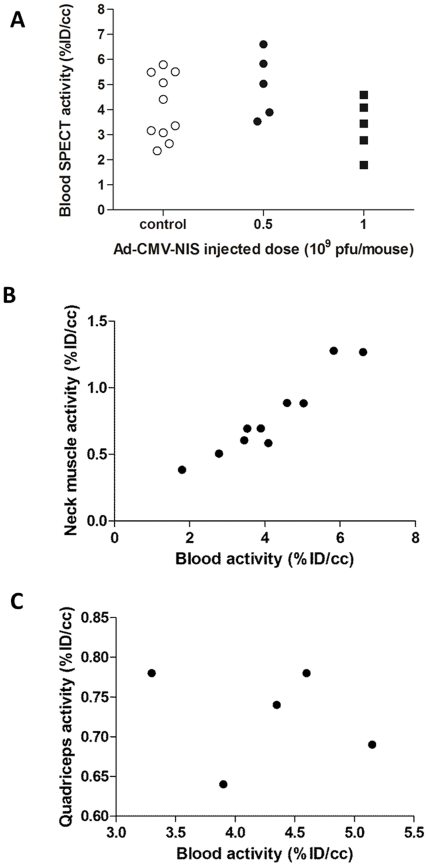
Comparison of blood SPECT activity in control and Ad-CMV-rNIS-injected mice. A. Balb/c mice were injected intravenously with saline buffer (empty circles), 5×10^8^ PFU (black circles) or 1×10^9^ PFU (squares) Ad-CMV-rNIS. Four days later, SPECT/CT imaging was performed. ROIs were drawn in the left ventricular cavity of the heart. The activity was calculated and converted to %ID/cc. B and C. Mice were scanned by SPECT/CT and the blood activity (measured as described above) was compared with the activity in the neck muscles (B) or in the quadriceps (C). For calculations of the activities in the muscles, a ROI of similar size to that used for the calculation of the blood activity was drawn and positioned on the muscles.

To determine the blood activity, and at each time-point, a 2-mm-diameter region of interest in the left ventricular cavity of the heart was drawn, using fused SPECT/CT images. Fig. 3 shows that the blood pertechnetate content varied greatly from one animal to another. Despite the fact that a strictly identical protocol was used for the whole study, the blood pertechnetate content varied between 2 %ID/cc and more than 6%ID/cc. Importantly, the comparison between blood content in adenovirus-injected (5×10^8^ and 1×10^9^ PFU) animals and non-injected controls did not change significantly, indicating that strong expression of NIS in the liver did not significantly affect the pertechnetate content of the blood (Fig. 3A). Regions of interests of similar sizes were drawn in the neck muscles and quadriceps of experimental animals and the activities were compared to blood activities. Fig. 3B shows a good correlation between neck muscle and blood activity (R^2^ = 0.90), while a poor correlation was observed between quadriceps and blood activity (R^2^ = 0.030, Fig. 3C).

### Correlation between 99mTcO4− uptake measured by SPECT/CT and by post-mortem β-counting of a biopsy

**Figure 4:**
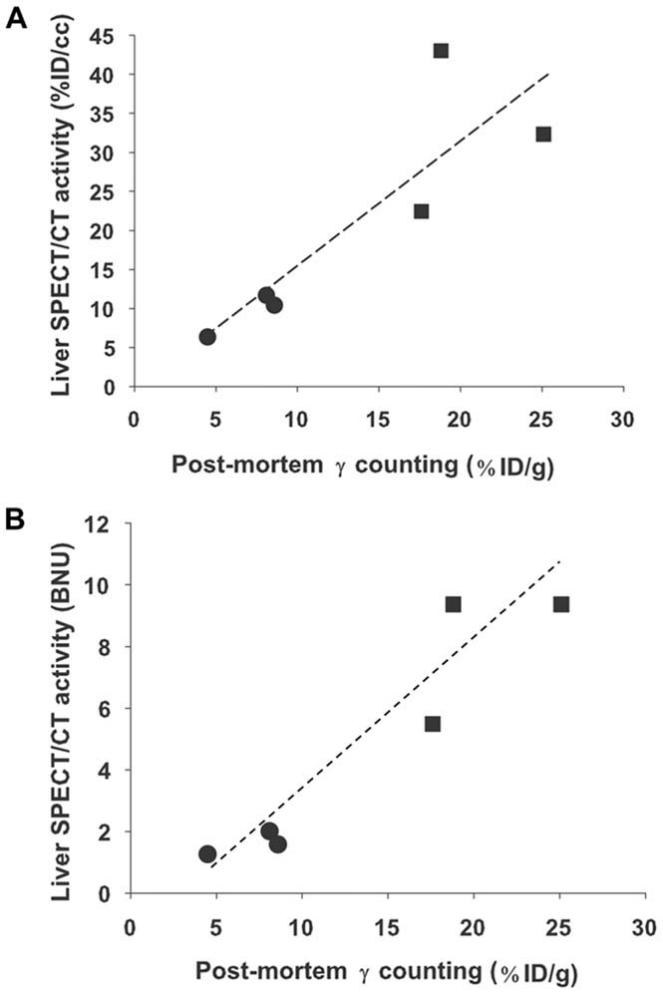
Comparison of the ^99m^TcO_4_^−^ uptake determined by *ex-vivo β*–counting of liver biopsies and measured by SPECT/CT. Balb/c mice were injected intravenously with either 5×10^8^ (circles) or 10^9^ PFU (squares) Ad-CMV-rNIS. Four days later, 100 MBq ^99m^TcO_4_^−^ were injected intraperitoneally and SPECT imaging was performed. Blood and liver activities were measured. After imaging, the radioactivity in the liver of each individual mouse was measured *ex vivo*. Values were expressed as (A) a percentage of the injected dose per volume (%ID/cc) or (B) normalised to blood activity.

**Figure 5:**
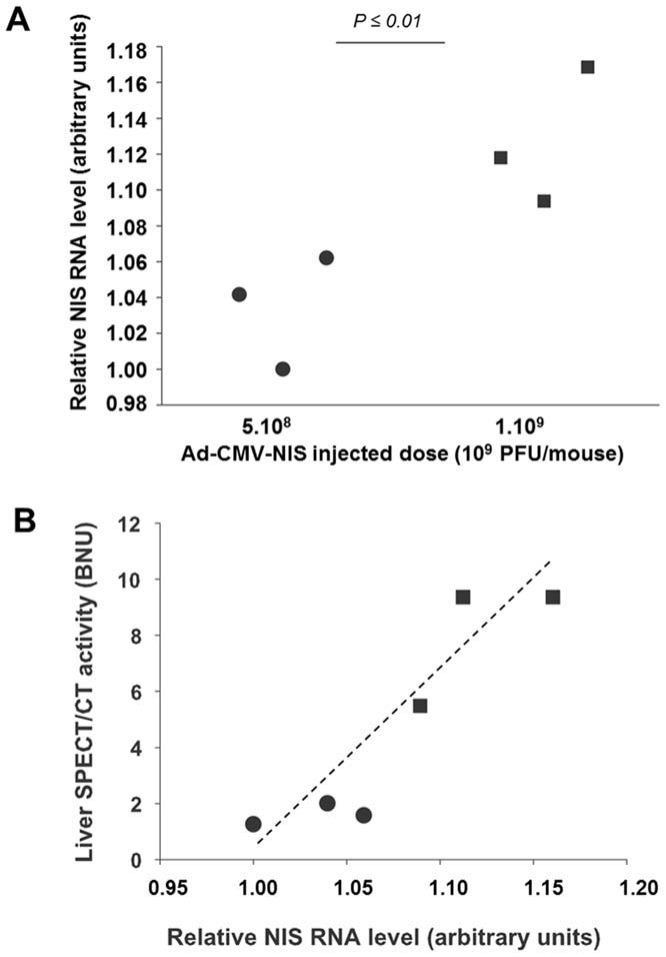
SPECT-imaging and quantitative RT-PCR analysis. (A) Quantitative RT-PCR analysis of NIS expression in the liver of adenovirus-injected mice. Total RNA was extracted from livers of mice intravenously injected with either 5×10^8^ (circles) or 1×10^9^ (squares) PFU Ad-CMV-rNIS, treated with DNase and reverse transcribed. Primers for murine GAPDH RNAs were used for normalisation and the lowest value was set as 1 arbitrary unit. ΔCt values were calculated by subtracting the Ct of the GAPDH housekeeping gene from the Ct of the targeted gene, measured in the same RNA preparation, and the lowest value was set as 1 arbitrary unit. (B) Comparison of the activity of adenovirus-transfected liver with the relative level of NIS mRNA. Balb/c mice were injected intravenously with either 5×10^8^ (circles) or 1×10^9^ PFU (squares) Ad-CMV-rNIS. Four days later, 100 MBq ^99m^TcO_4_^−^ were injected intraperitoneally and images were acquired on a SPECT/CT camera. ROIs were drawn in left and right liver lobes and the average activity was normalised to blood activity.

To establish whether the quantitative data obtained from SPECT/CT images are accurate, 6 animals were culled 3 days after virus injection, liver biopsies were collected, their radioactive content measured immediately after cull and compared with those calculated from SPECT/CT imaging. Fig. 4A shows a good correlation (R^2^ = 0.73) between activities measured by SPECT/CT imaging (expressed in %ID/cc) and post-mortem *β*-counting of biopsies. When the same comparison was performed using blood-normalised uptakes (BNU) (Fig. 4B), the correlation was even greater (R^2^ = 0.89). In separate animals administered with either 5×10^8^ (n = 3) or 1×10^9^ PFU (n = 3) Ad-CMV-rNIS, liver biopsies were collected to compare NIS gene expression and radiotracer accumulation measured by SPECT (Fig. 5). Quantitative RT-PCR analysis measuring NIS transcript levels showed that liver NIS expression increased with adenovirus dose administered (Fig. 5A) and that a good correlation (R^2^ = 0.83) could be established between the relative NIS expression levels and radiotracer uptake in the liver normalised to that of the blood, measured by SPECT/CT (Fig. 5B). When non-normalised data are analysed, an even greater coefficient of correlation is obtained (R^2^ = 0.93). Altogether, these results demonstrate that SPECT can provide quantitative information on the level of ectopic NIS expression in the liver. When the dataset is analysed using statistics, the normalisation to blood activity provides accurate data but this normalisation is not an absolute requirement.

### Long-term kinetic study after adenoviral vector-mediated gene transfer to the liver

**Figure 6:**
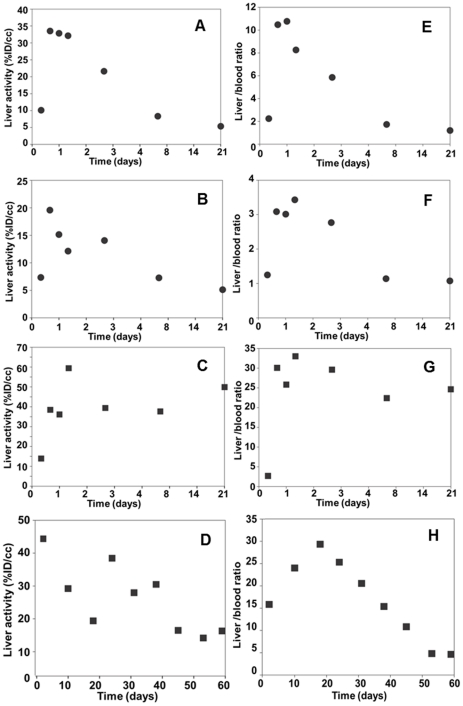
Monitoring of NIS gene transfer in the liver after systemic Ad-CMV-rNIS administration. Balb/c mice were injected intravenously with either 5×10^8^ (A, E, B, F) (n = 2) or 1×10^9^ PFU (n = 2) (C, D, G, H) Ad-CMV-rNIS. At different time-points after adenoviral administration, 100 MBq ^99m^TcO_4_^−^ were injected intraperitoneally and SPECT/CT imaging was performed. ROIs were drawn in left and right liver lobes. The average activity was calculated and converted to percent of the injected dose per centimetre cubed (%ID/cc) (column 1, A–D). These values were normalised with the blood (column 2, E–H) activities. In each graph, the data obtained on individual animals were plotted.

Mice administered with either 5×10^8^ or 1×10^9^ PFU Ad-CMV-rNIS were scanned serially. For each experimental point, ROIs of 4 mm diameter were drawn in ‘representative’ areas of the right and left lobes of the liver and the average values were used to calculate the %ID/cc at each time-point. The kinetics presented in Figs. 6A and 6B show that, upon administration of 5×10^8^ PFU Ad-CMV-rNIS, radiotracer uptake reaches its maximal level between 48 h and 3 d after virus injection and decreases afterwards to reach a near-basal level by day 21. By contrast, upon injection of 1×10^9^ PFU virus, a near-maximum radiotracer uptake is still observed in the liver 21 d after virus administration (Figs. 6C and 6D) and the near-basal level is only reached after 60 d (Fig. 6D). Overall, these kinetics are in good agreement with previous studies [33]. This overall trend is observed in all experimental animals but the kinetics in individual animals showed rather incoherent results, at specific time-points (Figs. 6B, 6C, 6D). Activities in the liver increased, decreased and increased again in a manner not compatible with reported studies of liver transduction with a replication-deficient adenovirus [33], suggesting an experimental or methodological bias. These inconsistencies were not linked with the choice of ROI, as different ROIs positioned differently in the liver produced a similar dataset (not shown).

A possible explanation for the apparently incoherent liver uptake of ^99m^TcO_4_^−^ in Ad-CMV-rNIS-transduced mice (Figs. 6B, 6C, 6D) could be the variability in the ^99m^TcO_4_^−^ blood content demonstrated in Fig. 3. To address this question, and at each time-point, the liver activity was normalised to that of the blood. The results presented in Figs. 6E, 6F, 6G and 6H show a much more coherent dataset than without the blood correction.

### Imaging of NIS-expressing cells

**Figure 7:**
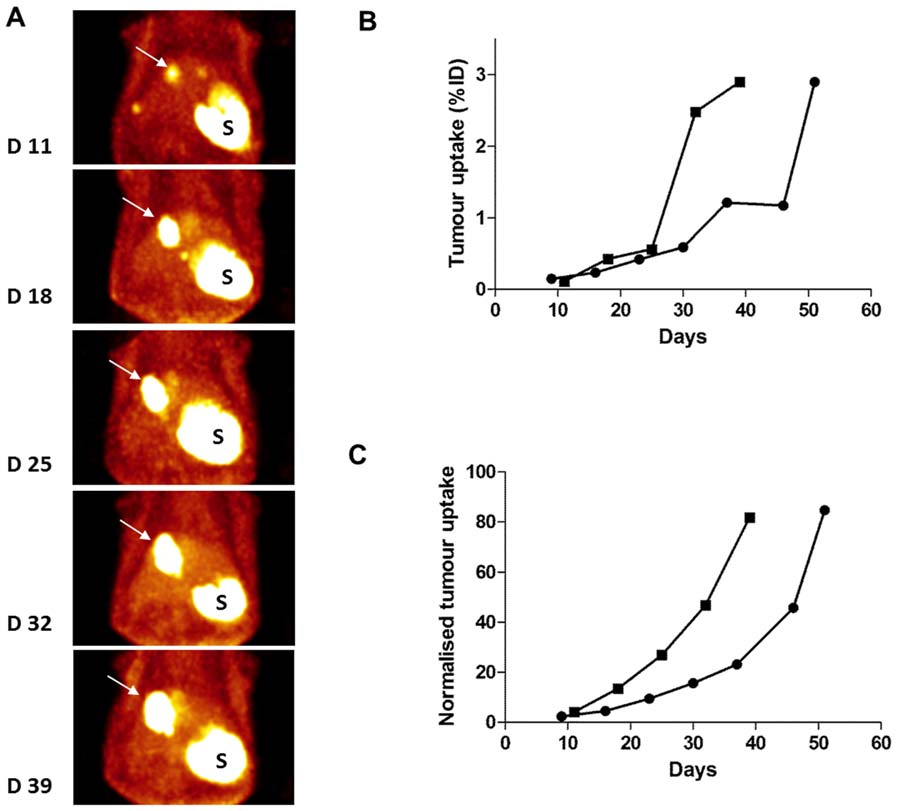
Non-invasive monitoring of colorectal cancer liver metastases. (A) Volume-rendering images of a mouse bearing liver HT29-NIS tumours (white arrow). The kinetics of tumour growth were monitored by SPECT imaging at days 11, 18, 25, 32 and 39 after injection of 2×10^6^ HT29-NIS cells in the liver. Tracer also accumulated in the stomach (S). (B–C) Time course of ^99m^TcO_4_^−^ accumulation in liver metastases as determined by serial scanning. From each SPECT/CT image acquired in (A), a three-dimensional ROI was drawn around the liver tumour using a threshold of 50% of the maximal activity. The counts in the ROI were calculated and expressed as (B) a percentage of the injected dose (%ID) accumulated in liver tumour nodules or (C) normalised to the blood activity. Data of two individual animals are presented.

HT29-NIS cells were injected in the liver of SCID mice and pertechnetate uptake was determined by SPECT/CT imaging. Fig. 7A shows the volume rendering of the pertechnetate uptake by hepatic tumours, at different times after HT29-NIS administration. The quantitative analyses were performed by drawing regions of interest around the tumours and taking into account the top 50% of the activity in the tumour. On one mouse, the representation of data as %ID/cc showed a first phase of tumour growth up to day 25 and then a sudden 5-fold increase in the capability of the tumour to capture pertechnetate on day 32 (square on Fig. 7B). This sudden jump is inconsistent with the visual comparison of the images at days 25 and 32 (Fig. 7A). Normalisation of tumour to blood activities (square on Fig. 7C) produced a dataset consistent with the volume-rendering images presented in Fig. 7A. A very similar smoothing effect of the blood normalisation was observed on a second animal (presented as circles in Fig. 7B and C). The non-normalised data indicate a stagnation in tumour growth between days 37 and 46 after tumour-cells inoculation, while normalisation to blood activity shows a constant increase in tumour size, more consistent with the growth, *in vivo*, of HT29 tumour cells.The smoothing effect of normalisation was observed on a total of 5 different mice (not shown).

## Discussion

In the present study, we highlight a problem which is likely to be encountered when data obtained from gene expression imaging analysis using the NIS reporter system are presented simply as a percentage of the injected dose, or even as %ID/cc. Variation in pertechnetate uptake levels by NIS expressing cells (either upon administration of a gene-delivery vector or after *ex-vivo* genetic engineering) can lead to inconsistencies with the subjective, visual observation of the images, and false interpretations. The impact of this problem can be reduced, or even removed, when data obtained on a cohort of animals are pooled and analysed statistically (see dataset and comments of Figure 5). However, when the imaging dataset obtained from individual animals are observed separately (in long-term kinetics, for example), the ‘statistical smoothing’ does not occur and great variability, and even experimental inconsistencies, may be reported inadvertently. These ‘noisy data’ may be attributed to the chaotic nature of the phenomenon observed, while they are in fact largely due to a methodological bias.

This problem is due to the fact that, upon administration of the radiotracer, and despite following rigorously a standard operating procedure, the concentration of the radiotracer in the blood varies greatly from one individual to another and even within the same individual when imaging is performed serially over a period of a few days/weeks. Our analysis on more than twenty different experimental points shows that a three-fold variation in the blood activity can be found (Fig. 3A). Attempts to reduce this variability by increasing or decreasing the length of time between radiotracer administration and the beginning of the scan did not provide any improvement (data not shown). On the contrary, our dataset tends to suggest that reducing the time between radiotracer injection and beginning the scan leads to an increased variability (data not shown). The measurement of muscle activity is an option that may be envisaged as an alternative (and sometimes more accessible measure than blood activity) to normalise data. However, if neck muscle activity appears to be well-correlated to blood activity (R^2^ = 0.90), quadriceps activity is not (R^2^ = 0.30). These observations suggest that only of subset of muscles are suitable for normalisation and that blood normalisation is the most reliable way to normalise datasets. In addition, our data suggest that normalisation of an ectopic expression of the NIS gene in the leg muscle should be performed using the activity in the non-transduced muscle.

The problem of radiotracer availability in the field of gene expression imaging has already been highlighted by others using, for example, a mutant *Herpes simplex* virus-1 thymidine kinase as a reporter gene [35], but it is further emphasised by the specificity of NIS-imaging: the radiotracer is not trapped inside the NIS-expressing cells, its intra-cellular concentration being directly dependent on its extracellular concentration. As a result, any variation in the tracer plasma/blood concentration is likely to have a dramatic effect on the quantitative data. This variability has also been highlighted in humans in another context in which NIS-mediated uptake of radio-iodide is a key factor: the radioiodine ablation of remnant thyroid tissue [31], [32], [36]. Hanscheid et al. [31] compared the thyroid remnant uptake after thyroid hormone withdrawal and after administration of recombinant human TSH. The difference in uptake between the two conditions disappeared when the target activity was normalised to the residence time in the blood in individual patients. This phenomenon is mainly related to the different rate of renal clearance in the two situations. For a given transfer coefficient, the target tissue uptake depends almost linearly on the blood activity, which is inversely proportional to the rate of renal clearance. Similarly, Verbug et al. [32] showed that absorbed dose in the blood is a better predictor of ablation success than administered activity. This is explained by the impact of renal clearance. If the clearance is lower, the target tissue will have a considerably higher amount of circulating iodide at its disposal, resulting in an increased efficiency of the treatment. In this latter, retrospective study involving 449 patients, the injected dose of ^131^I required to reach the critical blood dose of 350 mGy varied from 1 GBq to 7 GBq, with the vast majority of patients reaching this blood dose with an injected dose varying from 2 GBq to 5 GBq [32]. This dataset demonstrates that the variability in blood iodide or pertechnetate concentration is a phenomenon shared by mice and humans and that the mode of administration (intraperitoneal in our study in mice versus oral in humans) has no effect on this variability. In mice, the anaesthesia is probably amplifying this variability by affecting differentially the hemodynamic parameters of the experimental animals [37], [38].

To overcome the quantification problems caused by the variation in blood radiotracer concentration, we propose to normalise the data. The ratio of the accumulation of radiotracer in the transduced organ to that in a non-transduced organ, taken as background, has been proposed and used [29] without any formal validation. Alternatively, the thyroid gland could be chosen as a reference. In the course of the present study, we assessed whether thyroid activity could be used as an alternative reference to blood activity. For each experimental animal, regions of interest of 1 mm diameter were drawn around the left and right thyroid glands and the average activity of the whole gland was calculated. The activity in the liver was then divided by that of the thyroid gland and our data show that, as for ‘blood activity correction’, normalisation of liver activity by thyroid activity provides a much more coherent dataset (not shown). However, considering the difference in organification of pertechnetate and iodide by the thyroid [39], this organ may not be a ‘universal standard’ in NIS-imaging normalisation. By contrast, and considering that NIS-mediated uptake and concentration of iodide or pertechnetate is crucially dependent on the extracellular concentration of these anions, we advocate that, when possible, the blood activity should be determined and used to normalise the activity measured in the organ/region of interest that expresses NIS ectopically. Considering that NIS-imaging has now reached the clinical setting in the context of cancer gene therapy [24], [25] and that recent pre-clinical studies have demonstrated the potential of the methodology in the field of cell therapy, this normalisation may be essential in order to obtain accurate and predictive information in future clinical studies in biotherapy.

## Materials and Methods

### Adenovirus and NIS-expressing cell line

The replication-incompetent adenovirus, Ad-CMV-rNIS, in which the immediate-early promoter of CMV drives the expression of rat NIS, has been described previously [40]. This virus was produced and titrated at the “Plateforme de production de vecteurs pré-cliniques du CHU de Nantes”, using a standard protocol. The HT-29 cell line (HTB-38, ATCC) was transfected with pcDNA3.1-mNIS (murine NIS) using the FuGENE 6 reagent (Roche) according to the manufacturer's instructions. Stable clones were selected by adding 1 mg/ml geneticin (G418) to the medium 3 d after transfection. Demonstration of NIS expression in stably transfected clones was performed by ^125^I uptake, western blot analysis and immunostaining experiments, showing a strong localisation of the NIS protein at the plasma membrane. One clone (HT29-NIS) was selected for *in-vivo* imaging experiments.

### Animal studies

Animal housing and procedures were conducted according to the guidelines of the French Agriculture Ministry and were approved by the local ethics committee. Gene-transfer studies were performed on female Balb/c mice obtained at 8 weeks of age from Janvier (Le Genest Saint Isle, France). Ad-CMV-rNIS (5×10^8^ or 1×10^9^ PFU/mouse) in sterile saline buffer (final volume, 200 µl) was administered intravenously. Control animals were injected with 200 µl saline buffer. For the induction of hepatic tumours, 2×10^6^ HT29-NIS were injected under the liver capsule of anaesthetised, 7-week-old SCID mice (Harlan, Gannat, France).

### MicroSPECT/CT studies

Although thyroxin is used in some studies to reduce or block the uptake of iodide or perctechnetate by the thyroid, all the experiments in this study were performed on animals not treated with thyroxin. At various times after adenovirus administration or tumour-cell injection, mice were injected intraperitoneally with 100 MBq ^99m^Tc pertechnetate (^99m^TcO_4_^−^) obtained from a freshly eluted ^99^Mo/^99m^Tc generator. Precisely 20 min later, mice were imaged under Isofluran anaesthesia (Baxter, Aerane). SPECT/CT scans were performed using a micro-SPECT-CT (eXplore speCZT CT120, General Electric), using a previously published protocol [9], [23].

Image analyses and quantitative determinations were performed using the ‘AMIDE’ software [41]. For quantification, three-dimensional regions of interest (ROIs) were outlined on each left and right liver lobe/thyroid glands (4 mm and 1 mm diameter, respectively). For blood content determination, a 2 mm diameter ROI was drawn in the left ventricular cavity of the heart. This method was used to calculate blood activity in all the mice of the study. The voxel content was calculated and converted to percent injected dose per cubic centimetre (%ID/cc). Values were then corrected for decay of the radioisotope, taking 6 h as the half-life of ^99m^TcO_4_^−^.

### β-counting of liver biopsies

Livers were recovered after cull and weighed. Radioactivity in the organs was measured using a Medi404-calibrated dose calibrator (Medisystem). Values were expressed as percentage of the injected dose per gram of tissues (%ID/g) after correcting for activity decay to the time of image acquisition. All activity values were expressed with reference to the beginning of SPECT imaging 20 min after pertechnetate injection.

### TaqMan real-time PCR experiments

Total RNA from the livers of mice injected intravenously with either 5×10^8^ or 1×10^9^ PFU Ad- CMV-rNIS was extracted using Nucleospin RNAII (Machereyl-Nagel, France) and transcribed into cDNA using the Superscript III enzyme (Invitrogen, France). Real-time PCR was performed with the 7900HT Fast Real-Time PCR System and carried out using TaqMan® gene expression assays (Applied Biosystem, France). Primer sets were designed by, and purchased from, Applied Biosystems. The sequence of the primers is not available but the primers can be purchased form Applied Biosystem, using the following reference numbers: Rn 00583900-m1 for NIS and Mm-013518-11 for GAPDH. Cycle parameters were 95°C for 20 s followed by 40 cycles of 95°C for 1 s and 60°C for 20 s. Relative mRNA expression levels were determined using ΔCt values obtained by subtracting Ct control (mouse GAPDH) from Ct target gene (rat NIS), measured in the same RNA preparation.

### Immunohistochemistry

After culling the animals, livers were dissected, paraffin embedded and cut into 4-µm-thick sections. The paraffin was then removed and the sections were rehydrated and subjected to an antigen-retrieval treatment with a solution of citrate buffer, pH 6, using an automate (PT Link, Dako). Immunostaining was performed following a standard protocol (Dako EnVisionTM FLEX using an automated immunostainer, Autostainer, Dako). Endogenous peroxide was blocked using the EnVisionTM FLEX Peroxidase-Blocking Reagent. After pre-treatment, slides were incubated for 20 min at room temperature with a rabbit polyclonal antibody against NIS (antibody 25, see [42]) at a 1∶200 dilution. rNIS immunostaining was performed with a secondary antibody anti-mouse and rabbit/HRP (Dako, DM822) using a 3,3′ –diaminobenzidine (DAB) co-substrate [42]. The DAB-stained sections were counterstained with Harris haematoxylin (Sigma, Saint Quentin Fallavier, France). Image acquisition was performed using a Nikon 80i microscope equipped with a DS-5M-L1 digital camera.

### Statistical analysis

Statistical analysis was performed using Prism (GraphPad software). Dual comparisons were made using the student t-test and comparisons between multiple conditions were analysed using ANOVA. Statistical significance was set at P<0.05.

## References

[1] Hingorani M, Spitzweg C, Vassaux G, Newbold K, Melcher A (2010). The biology of the sodium iodide symporter and its potential for targeted gene delivery.. Curr Cancer Drug Targets.

[2] Spitzweg C, Joba W, Eisenmenger W, Heufelder AE (1998). Analysis of human sodium iodide symporter gene expression in extrathyroidal tissues and cloning of its complementary deoxyribonucleic acids from salivary gland, mammary gland, and gastric mucosa.. J Clin Endocrinol Metab.

[3] Perron B, Rodriguez AM, Leblanc G, Pourcher T (2001). Cloning of the mouse sodium iodide symporter and its expression in the mammary gland and other tissues.. J Endocrinol.

[4] Baril P, Martin-Duque P, Vassaux G (2010). Visualization of gene expression in the live subject using the Na/I symporter as a reporter gene: applications in biotherapy.. Br J Pharmacol.

[5] Chisholm EJ, Vassaux G, Martin-Duque P, Chevre R, Lambert O (2009). Cancer-specific transgene expression mediated by systemic injection of nanoparticles.. Cancer Res.

[6] Klutz K, Russ V, Willhauck MJ, Wunderlich N, Zach C (2009). Targeted radioiodine therapy of neuroblastoma tumors following systemic nonviral delivery of the sodium iodide symporter gene.. Clin Cancer Res.

[7] Watanabe Y, Horie S, Funaki Y, Kikuchi Y, Yamazaki H (2010). Delivery of Na/I symporter gene into skeletal muscle using nanobubbles and ultrasound: visualization of gene expression by PET.. J Nucl Med.

[8] Merron A, Baril P, Martin-Duque P, de la Vieja A, Tran L (2010). Assessment of the Na/I symporter as a reporter gene to visualize oncolytic adenovirus propagation in peritoneal tumours.. Eur J Nucl Med Mol Imaging.

[9] Merron A, Peerlinck I, Martin-Duque P, Burnet J, Quintanilla M (2007). SPECT/CT imaging of oncolytic adenovirus propagation in tumours in vivo using the Na/I symporter as a reporter gene.. Gene Ther.

[10] Dingli D, Kemp BJ, O'Connor MK, Morris JC, Russell SJ (2006). Combined I-124 positron emission tomography/computed tomography imaging of NIS gene expression in animal models of stably transfected and intravenously transfected tumor.. Mol Imaging Biol.

[11] Carlson SK, Classic KL, Hadac EM, Dingli D, Bender CE (2009). Quantitative molecular imaging of viral therapy for pancreatic cancer using an engineered measles virus expressing the sodium-iodide symporter reporter gene.. AJR Am J Roentgenol.

[12] Barton KN, Tyson D, Stricker H, Lew YS, Heisey G (2003). GENIS: gene expression of sodium iodide symporter for noninvasive imaging of gene therapy vectors and quantification of gene expression in vivo.. Mol Ther.

[13] Huang R, Zhao Z, Ma X, Li S, Gong R (2011). Targeting of tumor radioiodine therapy by expression of the sodium iodide symporter under control of the survivin promoter.. Cancer Gene Ther.

[14] Groot-Wassink T, Aboagye EO, Wang Y, Lemoine NR, Keith WN (2004). Noninvasive imaging of the transcriptional activities of human telomerase promoter fragments in mice.. Cancer Res.

[15] Chen L, Altman A, Mier W, Lu H, Zhu R (2006). 99mTc-pertechnetate uptake in hepatoma cells due to tissue-specific human sodium iodide symporter gene expression.. Nucl Med Biol.

[16] Sieger S, Jiang S, Schonsiegel F, Eskerski H, Kubler W (2003). Tumour-specific activation of the sodium/iodide symporter gene under control of the glucose transporter gene 1 promoter (GTI-1.3).. Eur J Nucl Med Mol Imaging.

[17] Higuchi T, Anton M, Dumler K, Seidl S, Pelisek J (2009). Combined reporter gene PET and iron oxide MRI for monitoring survival and localization of transplanted cells in the rat heart.. J Nucl Med.

[18] Higuchi T, Anton M, Saraste A, Dumler K, Pelisek J (2009). Reporter gene PET for monitoring survival of transplanted endothelial progenitor cells in the rat heart after pretreatment with VEGF and atorvastatin.. J Nucl Med.

[19] Terrovitis J, Kwok KF, Lautamaki R, Engles JM, Barth AS (2008). Ectopic expression of the sodium-iodide symporter enables imaging of transplanted cardiac stem cells in vivo by single-photon emission computed tomography or positron emission tomography.. J Am Coll Cardiol.

[20] Jung KH, Paik JY, Lee YL, Lee YJ, Lee J (2009). Trypsinization severely perturbs radioiodide transport via membrane Na/I symporter proteolysis: implications for reporter gene imaging.. Nucl Med Biol.

[21] Ricci D, Mennander AA, Pham LD, Rao VP, Miyagi N (2008). Non-invasive radioiodine imaging for accurate quantitation of NIS reporter gene expression in transplanted hearts.. Eur J Cardiothorac Surg.

[22] Goel A, Carlson SK, Classic KL, Greiner S, Naik S (2007). Radioiodide imaging and radiovirotherapy of multiple myeloma using VSV(Delta51)-NIS, an attenuated vesicular stomatitis virus encoding the sodium iodide symporter gene.. Blood.

[23] Peerlinck I, Merron A, Baril P, Conchon S, Martin-Duque P (2009). Targeted radionuclide therapy using a Wnt-targeted replicating adenovirus encoding the Na/I symporter.. Clin Cancer Res.

[24] Barton KN, Stricker H, Brown SL, Elshaikh M, Aref I (2008). Phase I study of noninvasive imaging of adenovirus-mediated gene expression in the human prostate.. Mol Ther.

[25] Barton KN, Stricker H, Elshaikh MA, Pegg J, Cheng J (2011). Feasibility of Adenovirus-Mediated hNIS Gene Transfer and (131)I Radioiodine Therapy as a Definitive Treatment for Localized Prostate Cancer.. Mol Ther.

[26] Rajecki M, Kangasmaki A, Laasonen L, Escutenaire S, Hakkarainen T (2011). Sodium Iodide Symporter SPECT Imaging of a Patient Treated With Oncolytic Adenovirus Ad5/3-Delta24-hNIS.. Mol Ther.

[27] Groot-Wassink T, Aboagye EO, Wang Y, Lemoine NR, Reader AJ (2004). Quantitative imaging of Na/I symporter transgene expression using positron emission tomography in the living animal.. Mol Ther.

[28] Carlson SK, Classic KL, Hadac EM, Bender CE, Kemp BJ (2006). In vivo quantitation of intratumoral radioisotope uptake using micro-single photon emission computed tomography/computed tomography.. Mol Imaging Biol.

[29] Siddiqui F, Barton KN, Stricker HJ, Steyn PF, Larue SM (2007). Design considerations for incorporating sodium iodide symporter reporter gene imaging into prostate cancer gene therapy trials.. Hum Gene Ther.

[30] Penheiter AR, Griesmann GE, Federspiel MJ, Dingli D, Russell SJ (2011). Pinhole micro-SPECT/CT for noninvasive monitoring and quantitation of oncolytic virus dispersion and percent infection in solid tumors.. Gene Ther.

[31] Hanscheid H, Lassmann M, Luster M, Thomas SR, Pacini F (2006). Iodine biokinetics and dosimetry in radioiodine therapy of thyroid cancer: procedures and results of a prospective international controlled study of ablation after rhTSH or hormone withdrawal.. J Nucl Med.

[32] Verburg FA, Lassmann M, Mader U, Luster M, Reiners C (2011). The absorbed dose to the blood is a better predictor of ablation success than the administered 131I activity in thyroid cancer patients.. Eur J Nucl Med Mol Imaging.

[33] Wu JC, Sundaresan G, Iyer M, Gambhir SS (2001). Noninvasive optical imaging of firefly luciferase reporter gene expression in skeletal muscles of living mice.. Mol Ther.

[34] Groot-Wassink T, Aboagye EO, Glaser M, Lemoine NR, Vassaux G (2002). Adenovirus biodistribution and noninvasive imaging of gene expression in vivo by positron emission tomography using human sodium/iodide symporter as reporter gene.. Hum Gene Ther.

[35] Richard JC, Zhou Z, Chen DL, Mintun MA, Piwnica-Worms D (2004). Quantitation of pulmonary transgene expression with PET imaging.. J Nucl Med.

[36] Hanscheid H, Verburg FA, Biko J, Diessl S, Demidchik YE (2011). Success of the postoperative (131)I therapy in young Belarusian patients with differentiated thyroid cancer after Chernobyl depends on the radiation absorbed dose to the blood and the thyroglobulin level.. Eur J Nucl Med Mol Imaging.

[37] Meneton P, Ichikawa I, Inagami T, Schnermann J (2000). Renal physiology of the mouse.. Am J Physiol Renal Physiol.

[38] Lorenz JN (2002). A practical guide to evaluating cardiovascular, renal, and pulmonary function in mice.. Am J Physiol Regul Integr Comp Physiol.

[39] Zuckier LS, Dohan O, Li Y, Chang CJ, Carrasco N (2004). Kinetics of perrhenate uptake and comparative biodistribution of perrhenate, pertechnetate, and iodide by NaI symporter-expressing tissues in vivo.. J Nucl Med.

[40] Faivre J, Clerc J, Gerolami R, Herve J, Longuet M (2004). Long-term radioiodine retention and regression of liver cancer after sodium iodide symporter gene transfer in wistar rats.. Cancer Res.

[41] Loening AM, Gambhir SS (2003). AMIDE: a free software tool for multimodality medical image analysis.. Mol Imaging.

[42] Dayem M, Basquin C, Navarro V, Carrier P, Marsault R (2008). Comparison of expressed human and mouse sodium/iodide symporters reveals differences in transport properties and subcellular localization.. J Endocrinol.

